# Supplementary material for: Complexome profiling of the Chlamydomonas psb28 mutant reveals TEF5 as an early PSII assembly factor
Source: Plant Cell. 2025 Mar 17;37(6):koaf055. doi: 10.1093/plcell/koaf055 (PMC12164590; doi:10.1093/plcell/koaf055)
Supplement: koaf055_Supplementary_Data [file koaf055_supplementary_data.zip › psb28_interactive.zip!/plots/intensity/qualityPlots/peptides/GHL1_Cre03.g171050.t1.1.html]

### Cre03.g171050.t1.1

Trivial name: GHL1  
SeqCoverage [median]: 7.30  
Mass: 57.13 kDa  
  
 

| Sequence | MissedCleavage | Mass [Da] | wtIntensity | muIntensity | Score |
| --- | --- | --- | --- | --- | --- |
| ALMIDSYMK | 0 | 1070.51 | 383.79 | 0.00 | 45.08 |
| IMDLVTAGR | 0 | 974.52 | 0.00 | 811.94 | 96.95 |
| LAADIGSTTFR | 0 | 1150.60 | 358.10 | 2470.41 | 82.75 |
| SFGVSAIR | 0 | 835.46 | 0.00 | 888.75 | 89.30 |
|  |  |  | 741.9 | 4171.1 |  |
